# Supplementary material for: Unveiling Genital Crohn’s Disease: Clinical Complications, Diagnosis, and Treatment, a Comprehensive Review of Case Reports
Source: Gastro Hep Adv. 2026 Mar 19;5(6):100918. doi: 10.1016/j.gastha.2026.100918 (PMC13187590; doi:10.1016/j.gastha.2026.100918)
Supplement: Supplementary Table 1 [file mmc1.pdf]

Supplementary Table 1: The distribution of the diagnostic modalities used across the included cases is summarized.

	Frequency	Percent
	1	2.0
barium meal	2	3.9
biopsy	12	23.5
blood/serology	1	2.0
clinical examination	1	2.0
colonoscopy	3	5.9
CT	10	19.6
endoscopy	4	7.8
MRI	12	23.5
radiographic	1	2.0
ultrasound/US	4	7.8
Total	51	100.0
