# Supplementary material for: Unveiling Genital Crohn’s Disease: Clinical Complications, Diagnosis, and Treatment, a Comprehensive Review of Case Reports
Source: Gastro Hep Adv. 2026 Mar 19;5(6):100918. doi: 10.1016/j.gastha.2026.100918 (PMC13187590; doi:10.1016/j.gastha.2026.100918)
Supplement: Supplementary Table 2 [file mmc2.pdf]

Supplementary Table 2: Differential Diagnosis of Vulvar Crohn's Disease and Its Common Mimics.

**Categoy**	**Condition**	**Clinical Features**	**Distinguishing Features**
**Inflammatory**	Behçet's disease	Bipolar aphthous ulcers (oral/genital), recurrent ulcers, arthralgia, uveitis	Pathergy test positive; systemic inflammatory involvement
	Hidradenitis suppurativa	Painful nodules, abscesses, sinus tracts, and scarring in intertriginous areas	Follicular plugging and suppurative granulomas
	Sarcoidosis	Non-tender papules/plaques,	Non-caseating granulomas; systemic

		systemic symptoms (e.g., cough, fatigue)	organ involvement
	Pyoderma gangrenosum	Painful ulcers with undermined violaceous borders; associated systemic disease	Ulcerative lesions without bacterial cause
	Psoriasis	Well-demarcated, erythematous plaques with scaling; pruritus	Auspitz sign; silvery scales; nail pitting
	Atopic/Contact dermatitis	Pruritic erythematous rash after irritant/allergen exposure	Vesicles in acute phase; history of exposure

**Infectious**	Vulvovaginitis (fungal: C. albicans, bacterial: G. vaginalis, parasitic: T. vaginalis)	Vaginal discharge, pruritus, erythema, dysuria	Positive microscopy: yeast, clue cells, or motile trichomonads
	Other bacterial vulvitis (e.g., tuberculosis, actinomycosis, lymphogranuloma venereum)	Chronic non-healing ulcers, inguinal lymphadenopathy, systemic symptoms	Suggestive histology, specific bacterial culture or PCR
**Bacterial**	Tuberculosis (Mycobacterium tuberculosis)	Chronic non-healing ulcers, systemic symptoms (weight loss, fever)	Caseating granulomas on biopsy
	Staphylococcus aureus,		

	Streptococcus, Chlamydia trachomatis, Neisseria gonorrhoeae		
**Spirochete**	Syphilitic chancre (Treponema pallidum)	Solitary, painless ulceration; associated lymphadenopathy	Painless, clean-based ulcer with positive serology
**Viral**	Herpes simplex virus (HSV-2)	Painful vesicular lesions progressing to ulcers; recurrent episodes	Vesicles before ulceration; multinucleated giant cells (Tzanck)
	Ulcus vulvae acutum	Painful vulvar ulcers; viral prodrome (fever, URI symptoms)	Acute onset with systemic viral symptoms
	Condyloma acuminata (HPV)	Exophytic, wart-like papules/plaques; may be painful or	HPV-related histology: koilocytes and

		asymptomatic	papillomatosis
**Fungal**	Candidiasis	Erythematous rash, white plaques, satellite lesions, pruritus	Yeast forms and pseudo hyphae under microscopy
**Nutritional**	Zinc deficiency	Painful fissures, ulcers, poor wound healing; alopecia	Responds to zinc supplementation
**Malignant**	Squamous cell carcinoma	Chronic non-healing ulcer with indurated margins	Atypical squamous cells; invasive malignancy
	Vulvar intraepithelial	Pruritic, white/red patches or lesions	

	neoplasia		Dysplasia in epithelial layer
	Paget's disease of the vulva	Erythematous, eczematous lesions; chronic, pruritic, and non-responsive to therapy	Paget cells: large pale cells in epidermis
**Other**	Genital lymphoedema	Chronic swelling, thickened skin, recurrent infections	History of trauma, obesity, or lymphatic obstruction
	Foreign-body reaction	Ulceration, granulomas, and inflammation; history of foreign material exposure	Foreign material identified in granulomas
	Vulvar edema	Swelling, thickened	History of radiotherapy,

	(post-radiotherapy, lymphedema, anasarca)	skin, peau d'orange appearance	lymphatic obstruction, systemic edema
	Epidermoid carcinoma	Chronic ulceration or mass; pain; induration; bleeding	Atypical squamous cells, invasion into deeper tissue
