# Supplementary material for: Unveiling Genital Crohn’s Disease: Clinical Complications, Diagnosis, and Treatment, a Comprehensive Review of Case Reports
Source: Gastro Hep Adv. 2026 Mar 19;5(6):100918. doi: 10.1016/j.gastha.2026.100918 (PMC13187590; doi:10.1016/j.gastha.2026.100918)
Supplement: Supplementary Table 3 [file mmc3.pdf]

Supplementary Table 3: The frequency and percentage distribution of the various treatment modalities used in the reviewed cases are summarized.

	Frequency	Percent
adalimumab	3	6.7
antibiotics	5	11.1
azathioprine	7	15.6
CO2 vaginal laser	2	4.4
corticosteroids	2	4.4
infliximab	7	15.6
methotrexate	2	4.4
metronidazole	6	13.3
oral steroids	3	6.7
piperacillin	2	4.4
prednisolone	4	8.9
tazobactam	2	4.4
Total	45	100.0
