# Supplementary material for: Unveiling Genital Crohn’s Disease: Clinical Complications, Diagnosis, and Treatment, a Comprehensive Review of Case Reports
Source: Gastro Hep Adv. 2026 Mar 19;5(6):100918. doi: 10.1016/j.gastha.2026.100918 (PMC13187590; doi:10.1016/j.gastha.2026.100918)
Supplement: Supplementary Table 4 [file mmc4.pdf]

Supplementary Table 4: A summary of treatment options

Medication Class	Drug	Dosage/Regimen	Notes
Corticosteroids	Topical Fluocinolone	Twice daily for two weeks	Combined with adalimumab (40 mg) for symptom relief. Limited evidence suggests mixed efficacy; one study reported no improvement, while another noted symptom relief when combined with systemic therapy.
	Topical Clobetasol	Applied once or twice daily (exact frequency not specified)	Limited efficacy; gastrointestinal symptoms flared within one month
	Oral Prednisolone	60 mg daily (monotherapy)	Complete resolution reported in one study, though rare.
	Oral Prednisolone	50–200 mg/day	Initial symptom resolution was observed, but 72% of patients experienced relapse during dose tapering.

	Oral Prednisolone	40 mg daily	Often combined with immunomodulators (e.g., methotrexate 7.5 mg weekly, ustekinumab, or adalimumab) for enhanced efficacy in refractory cases.
	IV Prednisolone	High-dose IV (exact dose not specified)	Transitioned to oral prednisolone (30 mg daily) over two months, maintaining clinical remission.
TNF-α Inhibitors	Infliximab	Induction: 5 mg/kg IV at weeks 0, 2, and 6; Maintenance: 10 mg/kg every 8 weeks	Combined with carbon laser therapy in some cases. Demonstrated complete symptom resolution in patients with refractory disease.
	Adalimumab	40 mg weekly or every other week	Used alone or in combination with azathioprine (40 mg every other week during induction, 40 mg weekly as maintenance).
Immunos uppressan	Azathioprine	50–200 mg/day	Mean dose: 131.4 mg/day; treatment duration: 18.7 months (range: 5–46 months).

ts			Effective in combination with TNF-α inhibitors or corticosteroids.
	Methotrexate	7.5 mg weekly	Used in combination with oral prednisolone (40 mg daily) for refractory cases.
	Mycophenolate Mofetil	Usually discontinued due to side effects.	Not recommended due to lack of efficacy and adverse events in Crohn’s disease.
Aminosalicylates	Mesalazine	1 g three times daily	Only one study reported its use in combination with azathioprine, resulting in symptom resolution. Limited evidence for genital Crohn’s disease.
Biologic Therapies	Ustekinumab	Induction: 390 mg IV; Maintenance: 90 mg subcutaneously every 8 weeks	Used as co-therapy with oral prednisolone (40 mg daily) after failure of anti-TNF-α therapy. Led to almost complete resolution of symptoms.
Antibiotics	Doxycycline	100 mg once daily	Used for secondary infections (e.g., cellulitis,

			UTIs). Limited efficacy in treating primary genital Crohn's lesions.
	Co-trimoxazole	960 mg once daily or 480 mg twice daily	Commonly used for secondary infections.
	Co-amoxiclav	375 mg three times daily	Commonly used for secondary infections.
	Clindamycin	250 mg once daily	Commonly used for secondary infections.
	Trimethoprim	200 mg	Commonly used for secondary infections.
	Ciprofloxacin	500 mg twice daily	Commonly used for secondary infections.
	Piperacillin/Tazobactam	IV (combined with metronidazole)	Complete resolution of lesions reported in one case. Primarily used for severe infections.
Stem Cell Therapy	Autologous Stem Cells	Delivered via bioabsorbable fistula plug	Phase I trial (STOMP) showed complete clinical healing in 14/18 patients at 6 months and 13/17 at 12 months. MRI response observed in 12/18 patients at 6 months. Safe

			and effective for refractory cases.
Carbon Laser Therapy	Carbon Laser	Five sessions (frequency not specified)	Combined with infliximab (5 mg/kg induction, 10 mg/kg maintenance). Partial to complete symptom relief reported in some cases.
Surgical Interventions	Various Procedures	Detachment of inflamed bowel, fistula suturing, abscess drainage, debridement	Reserved for refractory cases or complications (e.g., abscesses, hypertrophic lesions). Extensive perineal debridement and deroofing to healthy tissue also reported, with good healing progress.
