# Supplementary material for: Unveiling Genital Crohn’s Disease: Clinical Complications, Diagnosis, and Treatment, a Comprehensive Review of Case Reports
Source: Gastro Hep Adv. 2026 Mar 19;5(6):100918. doi: 10.1016/j.gastha.2026.100918 (PMC13187590; doi:10.1016/j.gastha.2026.100918)
Supplement: Extended PDF [file mmc5.pdf]

Gastro Hep Advances 2026;5:100918

NARRATIVE REVIEWS

Unveiling Genital Crohn's Disease: Clinical Complications, Diagnosis, and Treatment, a Comprehensive Review of Case Reports

Bishoy Fahim,[1] Mohamed Elnaggar,[2] Mohamed Ayman Ebrahim,[3] Shady Sapoor,[4] Abdelrahman Helmy,[5] Mahmoud Abd El-Nasser,[6] Areeba Mariam Mehmood,[7] Safia Elshennawy,[8] Abdelrahman Sayed Al Komi,[9] Laith Shakharteh,[10] Mennatullah Ashour,[11] Ismail Elkhattib,[2] Mazen Gado,[5] Esraa Soliman,[12] Mohamed Abd El Aziz,[13] and Hassan Ghoz[14]

[1]Faculty of Medicine, Sohag University, Sohag, Egypt; [2]Hospital Medicine Department, Hartford Healthcare, Hartford, Connecticut; [3]Internal Medicine Department, Ascension Saint Joseph, Chicago, Illinois; [4]Faculty of Medicine, Benha University, Benha, Egypt; [5]Faculty of Medicine, Minia University, Minia, Egypt; [6]Faculty of Medicine, Kafr Elsheikh University, Kafr El-Shaikh, Egypt; [7]Department of Medicine, Faisal Masood Teaching Hospital, Sargodha, Pakistan; [8]Faculty of Medicine, Gastroenterology Department, Tanta University Hospitals, Tanta, Egypt; [9]Faculty of Medicine, Al-Azhar University, Qalubiya, Egypt; [10]The National Center for Diabetes, Endocrinology, and Genetics, University of Jordan, Amman, Jordan; [11]Faculty of Medicine, Benha University, Benha, Egypt; [12]Department of Gastroenterology and Hepatology, University of Nebraska Medical Center, Omaha, Nebraska; [13]Faculty of Medicine, Mansoura University, Mansoura, Egypt; and [14]Division of Gastroenterology and Hepatology, Department of Medicine, University of Maryland School of Medicine, Baltimore, Maryland

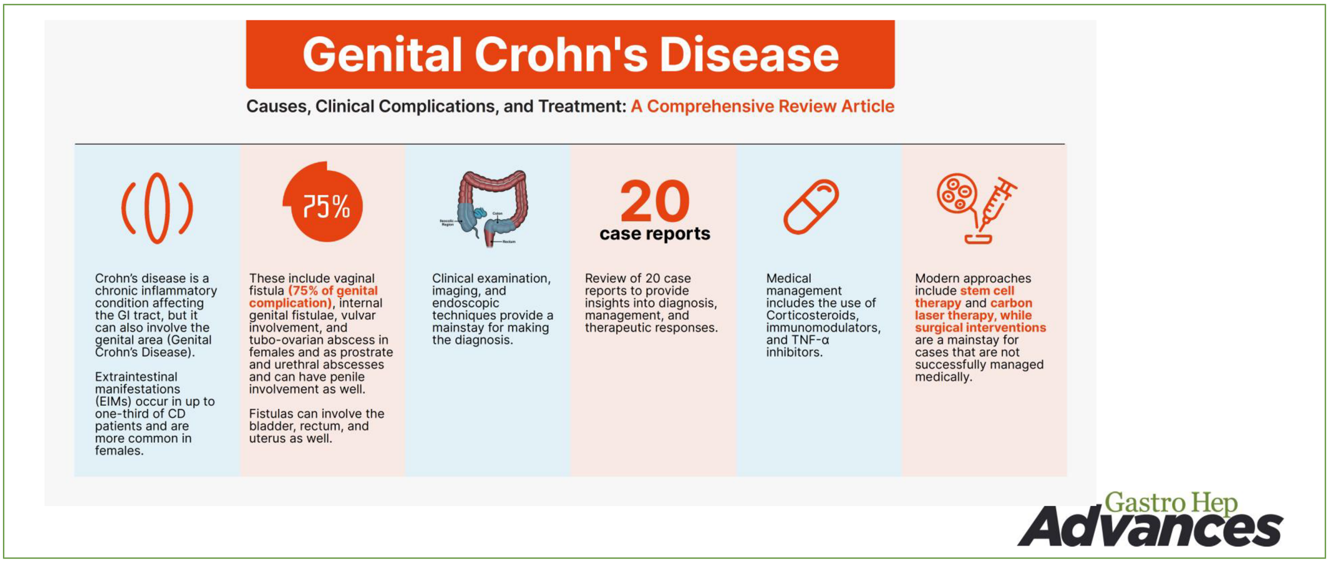

Genital Crohn's disease (GCD) is an infrequent extra-intestinal manifestation of Crohn's disease characterized by ulceration, pain, edema, and erythema in the anogenital area. GCD poses significant diagnostic and management challenges, mainly because there are no standardized clinical protocols. This review analyzed 20 case reports and series from 1983 to 2024, focusing on patients with GCD. The databases used for this search were PubMed, Scopus, and Google Scholar. The most common presentation in male patients was genital edema or swelling, seen in 88.9% of cases. Pain was the main presentation in 71.9% of cases among female patients. Several complications were reported, including rectovaginal and enterovaginal fistulas, tubo-ovarian abscesses, and penile involvement. The diagnosis was made based on clinical evaluation, which was supported by laboratory tests, physical examinations, imaging, and endoscopic procedures. Histological examinations of biopsied genital ulcers

Abbreviations used in this paper: CD, Crohn's disease; GCD, genital Crohn's disease; GI, gastrointestinal; MRI, magnetic resonance imaging; TNF-α, tumor necrosis factor-alpha.

Most current article

© 2026 The Authors. Published by Elsevier Inc. on behalf of American Gastroenterological Association Institute. This is an open access article under the CC BY license (http://creativecommons.org/licenses/by/4.0/).
2772-5723
https://doi.org/10.1016/j.gastha.2026.100918

demonstrated non-caseating granulomatous inflammation; however, this finding was observed in only 50% of patients. Moreover, pelvic magnetic resonance imaging is highlighted as a crucial diagnostic tool. The most common form of treatment is pharmacological therapy. Corticosteroids, immunosuppressants, and tumor necrosis factor-alpha inhibitors are the most frequently used drugs. A total of 14 studies reported that corticosteroids administered via intravenous and oral routes resulted in a notable reduction in disease severity. The tumor necrosis factor-alpha inhibitors adalimumab and infliximab showed notable efficacy. New therapies such as carbon laser therapy and autologous stem cell transplantation showed promising outcomes in refractory cases. Surgical procedures are reserved for refractory or complicated cases. This review highlights that medical management with corticosteroids, immunomodulators, and biologics remains the cornerstone of treatment for GCD, with surgery playing a role in refractory disease.

Keywords: Case Reports; Crohn's Disease; Genital Diseases

Introduction

Crohn's disease (CD) is a chronic, relapsing-remitting inflammatory disorder that can affect any part of the gastrointestinal (GI) tract, leading to a diverse range of symptoms and complications.[1] In addition to intestinal involvement, extraintestinal manifestations are observed in up to 33.3% of patients affecting various organ systems, including the skin, eyes, and joints.[2] Among these, genital Crohn's disease (GCD) represents a rare but significant manifestation, characterized by granulomatous inflammation of the genitalia and perineal region.[3] GCD may result from direct disease extension, fistulization between the intestine and genital structures, or may even occur independently of GI symptoms.[4–6]

Although GCD is more commonly reported in female patients,[7] its pathogenesis remains poorly understood, often leading to diagnostic delays and suboptimal management.[8] Clinical manifestations can vary widely, including genital edema, nodules, plaques, chronic suppuration, and abscess formation.[9] The involvement of genital organs, such as the vulva and perineum, is associated with significant morbidity and a profound negative impact on the quality of life.

The management of GCD is complex and often unpredictable, requiring a multidisciplinary approach.[8] Medical therapy remains the mainstay of treatment. It typically involves corticosteroids, immunomodulators, and tumor necrosis factor-alpha (TNF-α) inhibitors; however, refractory cases may necessitate surgical intervention. Emerging therapies such as carbon dioxide laser therapy and mesenchymal stem cell transplantation have demonstrated potential in selected cases.

This review aims to provide a comprehensive analysis of GCD by examining its clinical presentation, diagnostic challenges, disease course, and treatment strategies. By synthesizing findings from 20 case reports and series, this review enhances the understanding of this underrecognized manifestation of CD and highlights the evolving therapeutic landscape for its management.

Diagnosis

The diagnosis of GCD is based on clinical manifestations and a comprehensive approach, including serological testing to rule out infectious causes, physical examinations (gynecological and dermatological), imaging (ultrasonography, barium meal follow-through, pelvic magnetic resonance imaging [MRI], computed tomography scans, and MR enterography), and endoscopic procedures with biopsy. Biopsies from the skin, vulvar tissue, scrotal tissue, and bowel commonly reveal non-caseating granulomatous inflammation. Other investigations include stool calprotectin, urine analysis, and cultures.[10] The distribution of the diagnostic modalities used across the included cases is summarized in Supplementary Table 1; the percentages of the diagnostic methods used for GCD are presented in Figure 1.

Clinical Manifestations of Genital Crohn's Disease

GCD is a rare and challenging condition with distinct clinical presentations in both sexes. Genital lesions are the most common presentation of metastatic Crohn's disease in children. Understanding these differences is essential for accurate diagnosis and tailored treatment. The in-depth analysis of the clinical manifestations, based on the data provided in the included cases, is presented for male patients (Table 1) and female patients (Table 2); the clinical manifestations of GCD in males and females are presented in Figure 2.

Clinical Manifestations in Males

- Swelling and edema are the most common clinical finding, seen in 88.9% of patients. This is caused by the obstruction of the lymphatic ducts by granulation tissue formation. It could be generalized genital swelling or limited to a specific part of the male genitalia, such as the penis (66.7%) or scrotum (55.6%).
- Erythema was noted in 75% of patients, and it indicates localized inflammation or irritation.
- Urinary and sexual difficulties affect 69.4% of cases, mainly in older adults. These symptoms may include painful urination, difficulty in sexual performance, or related complications.
- Pneumaturia was reported in 22.2%, likely due to fistula formation affecting the urinary tract.
- Fever was present in 5.6%, particularly in children, suggesting a systemic inflammatory response.

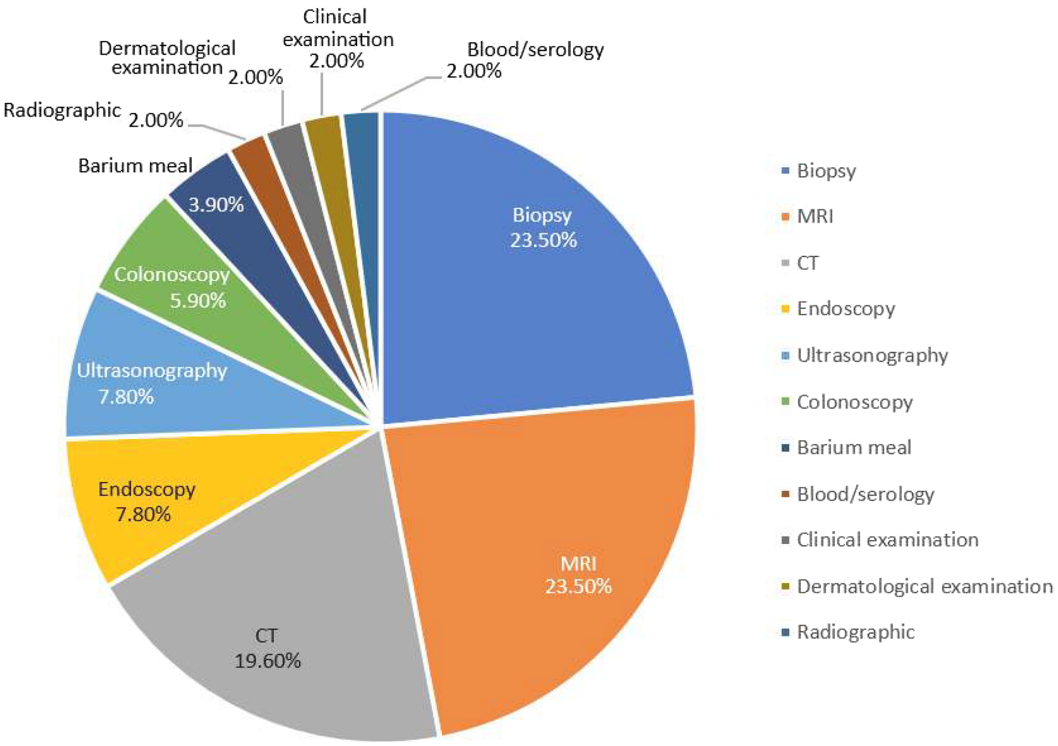

Figure 1. The percentages of the diagnostic methods used for GCD. CT, computed tomography.

- Purpura is a rare finding, observed in 2.8%.
- A scaly, raised rash occurs in 2.8% of patients; it is usually a nonpruritic, painless, and erythematous rash affecting the inguinal region, scrotum, and intergluteal region. This reflects a potential dermatological extension of the disease.

Clinical Manifestations in Females

- Tenderness and pain are the most common symptoms, unlike in male patients: pain is present in 71.9% of cases, significantly impacting the quality of life.
- Swelling and edema are found in 34.4%, which is less prevalent than in male patients.
- Erythema occurs in 15.6%, representing localized inflammation or redness.
- A burning sensation is a common complaint in 62.5% of cases.
- Passage of gas or feces from the vagina was reported in 62.5% of cases, typically due to rectovaginal fistulas.
- Foul-smelling vaginal discharge affects 65.6% of cases, often linked to infection or fistula.
- Painful vulvar ulcers are seen in 6.25% of cases, mainly in older adults, indicating severe localized tissue damage.
- Recurrent vulvar abscesses are rarely reported, occurring in 3.1%, and are associated with purulent vaginal discharge.
- Other rare manifestations include hyperkeratotic lesions found in 3.1%, which are seen in older female patients. Bilateral labial hypertrophy is another rare finding that occurs in 3.1% of patients; it is mostly seen in women of

Table 1. Clinical Manifestations in Males

Clinical feature	Male (n = 36)
Swelling/edema, no. (%)	32 (88.9)
Penile swelling/edema	24 (66.7)
Scrotal swelling/edema	20 (55.6)
Erythema, no. (%)	27 (75)
Urinary and sexual difficulties, no. (%)	25 (69.4)
Pneumaturia, no. (%)	8 (22.2)
Fever, no. (%)	2 (5.6)
Purpura, no. (%)	1 (2.8)
Scaly, raised rash, no. (%)	1 (2.8)

Table 2. Clinical Manifestations in Females

Clinical feature	Female (n = 32)
Swelling/edema, no. (%)	11 (34.4)
Erythema, no. (%)	5 (15.6)
Vaginal burning, no. (%)	20 (62.5)
Passage of gas or feces from the vagina, no. (%)	20 (62.5)
Foul-smelling vaginal discharge, no. (%)	21 (65.6)
Vaginal tenderness/pain, no. (%)	23 (71.9)
Painful vulvar ulcers, no. (%)	2 (6.25)
Recurrent vulvar abscess, no. (%)	1 (3.1)
Hyperkeratotic lesions, no. (%)	1 (3.1)
Bilateral labial hypertrophy, no. (%)	1 (3.1)
Pneumaturia, no. (%)	1 (3.1)

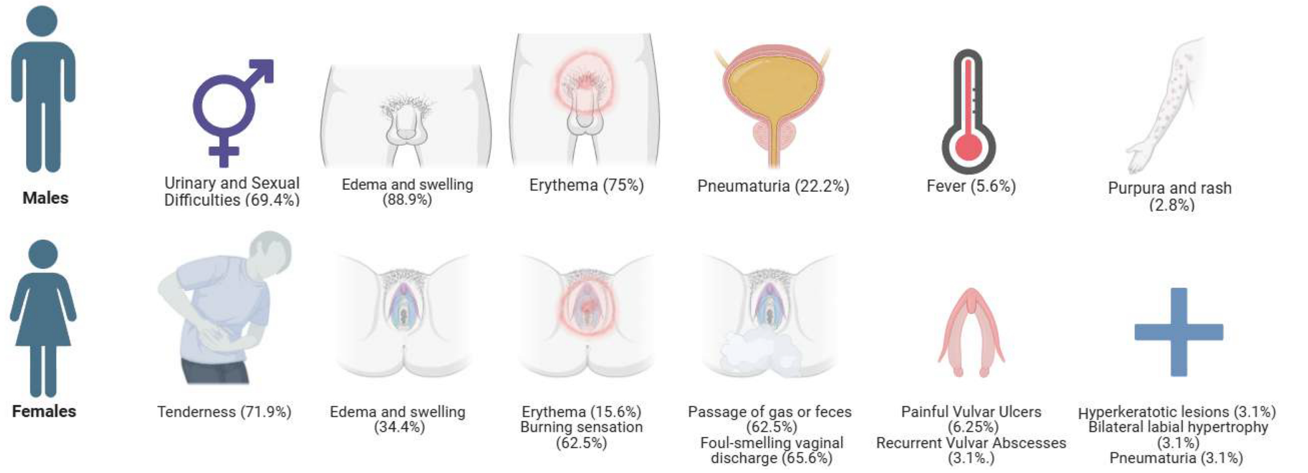

Figure 2. The clinical manifestations of genital Crohn's disease in males and females.

childbearing age. Pneumaturia was reported in 3.1%, likely linked to urinary fistula development.

Complications of Genital Crohn's Disease

Enterovaginal Fistula

Enterovaginal fistulae are defined as fistulas that arise from the ileum or colon. They appear to be associated with hysterectomy and often occur as de novo fistulae in women with CD. However, the potential surgical risks should not preclude timely intervention when indicated. Enterovaginal fistulas present with symptoms such as pelvic pain, dyspareunia, and vaginal discharge that contains Gram-negative organisms, which can mimic the signs of pelvic inflammatory disease and consequently lead to misdiagnosis. Additionally, anovaginal fistulas have been reported as a late complication following restorative proctocolectomy in patients diagnosed with CD. These fistulas require a multidisciplinary approach, including surgically trimming and closing the vaginal defects. A nutritional assessment is also required for an optimal outcome.[11]

Rectovaginal Fistulas

Rectovaginal fistulas were found to develop in 9% of women with anal CD. This type of fistula is reportedly more common in African American women than in Caucasian women. The horseshoe shape of these fistulae is distinctive; they start as anterior ulcers that erode into the vagina but can also begin as a posterior cryptoglandular opening that tracks to the vagina. The narrow tracts complicate diagnosis by direct examination. Therefore, examination under anesthesia, utilizing proctoscopy and vaginoscopy, plays a crucial role, allowing for simultaneous repair if needed. If endoscopy fails to identify a tract in symptomatic patients, methods such as a gastrografin enema or the methylene blue test (tampon test) can be employed. Symptoms in these fistulae may present more prominently than in others, as they are generally more challenging to manage medically. Treatment options include immunomodulators, intravenous cyclosporine, and infliximab, though results can be mixed. Data from a recent trial indicated a higher short-term closure rate of rectovaginal fistulas compared to a placebo.

Furthermore, maintenance treatments such as immunomodulators, intravenous cyclosporine, and infliximab were found to be more effective than placebo in sustaining fistula closure. In that trial, the closure rate was 61% at 10 weeks and 45% at 14 weeks, with 72% of responders no longer experiencing drainage by week 14. Another case reports a woman with a recurrent rectovaginal fistula who was treated successfully through a combination of autologous stem cell transplantation and surgical correction. The use of these techniques enabled the surgeon to overcome technical difficulties with postoperative healing. Mucosal advancement flaps were used to treat patients with trans-sphincteric and extrasphincteric fistula, with clinical improvement observed in 70% to 75% of patients. Neoplastic change is one of the most serious long-standing complications of fistulae, with mucinous adenocarcinoma being the most frequent type of neoplasm.[12]

Vulvar Involvement

Women with GCD commonly experience perineal and vulvar abnormalities, which may include local discomfort, pain, or dyspareunia. Physical findings such as labial swelling, erythema, tender deep ulcerations, nodular masses, or draining sinuses should be considered CD-related in the setting of chronic inflammatory bowel disease. Differential diagnosis of these manifestations includes Bartholin gland cysts, hidradenitis suppurativa, granulomatous diseases (Behcet and sarcoidosis), sexually transmitted infections (including syphilis, genital herpes, and lymphogranuloma venereum), or even sexual abuse. Vulvar CD most often originates as an extension from perianal and

anovaginal inflammatory changes. Metastatic CD, which refers to vulvar involvement noncontiguous to the GI tract, has been reported in both adults and children. It is rare and may precede or coexist with intestinal manifestations, making it a challenging diagnosis and a possibly unrecognized cause of vulvar pain. These inflammatory changes may appear as vulvar skin and subcutaneous thickening with T2-weighted hyperintense signal and positive contrast enhancement. Fistulae or abscess cavities are best captured using MRI. These findings should be reported when reviewing perianal or intestinal MRI studies in female patients with diagnosed or suspected GCD. Long-term conservative treatment with metronidazole alone or in combination with steroids is reported to be effective in treating clinical and imaging manifestations. However, advanced cases may require surgical vulvectomy.[11]

Tubo-Ovarian Abscess

CD could be complicated by a tubo-ovarian abscess, as demonstrated in a case report where a 16-year-old girl presented with suprapubic and right lower quadrant abdominal pain, fever, chills, and anorexia. A computed tomography scan revealed pelvic fluid collections and right ovarian inflammation. A complex right ovarian abscess was identified via exploratory laparoscopy. Treatment involved intravenous antibiotics, specifically doxycycline, gentamicin, cefotaxime, and metronidazole. Following this initial therapy, the patient continued with a 14-day regimen of oral doxycycline and metronidazole. Subsequently, a colonoscopy was performed, leading to a diagnosis of CD.[13]

Male Genital Involvement

Male patients with CD exhibit genital involvement much less often than female patients. However, abscess formation in the prostate gland and proximal urethra from direct extension of perianal inflammatory disease has been reported. The condition presents with local swelling or ulcerations. Pelvic–perianal MRI detects these involvements as fluid-filled, peripherally enhancing structures usually associated with perianal fistulas.

Penile Involvement

The development of fistulae is one of the complications associated with CD. Penetration of the scrotum, urethra, or penile shaft by a fistula can lead to penile involvement, which may manifest as a "watering-can" appearance during urination and result in subsequent urethral stricture. Treatment with azathioprine has been reported to yield a remarkable healing response for most fistulae and to enhance overall condition in such cases. However, a suprapubic cystotomy may be necessary in some cases for urinary drainage, as the penile lesion and urethral strictures persist.

Penile involvement may be a result of metastatic CD, which presents as a painless penile ulcer that consists of non-caseating granulomata. Topical steroid treatment could lead to nearly complete healing in such cases.[14]

Aseptic Abscess Syndrome

Aseptic abscess syndrome is a rare type of inflammatory disorder involving polymorphonuclear neutrophils, often associated with inflammatory bowel disease. A retrospective study included 71 patients, 37 of whom were male (52.1%).[15] The mean age at diagnosis was 34.5 $\pm$ 17 years. Of the total 71 cases, 5 (7%) were accompanied by genital involvement. Furthermore, 26 (36.6%) presented with CD. These interpretations suggest a significant relation between GCD and aseptic abscess syndrome, but further investigation is required to confirm this significance.

Differential Diagnosis of Genital Crohn's Disease

The differential diagnosis of GCD is broad and involves various inflammatory, infectious, and neoplastic conditions that can mimic its clinical presentation. Since many of these disorders present with similar features and require different therapeutic approaches, accurate diagnosis is essential. Variability in clinical presentation includes aphthous ulcerations, lymphedema, knife-cut ulcers, and perianal fistulae that may complicate diagnosis.[3] Histopathologic features, including non-caseating granulomas, intralymphatic granulomas, and chronic lymphocytic inflammation, are diagnostic hallmarks but may be absent in up to 50% of cases.[16,17] A comprehensive evaluation, including detailed history, physical examination, and appropriate laboratory and imaging studies, is crucial to distinguish GCD from similar diseases. The differential diagnoses are presented in Supplementary Table 2[3,10,18–23]; the genital Crohn's complications are presented in Figure 3.

Treatment for Genital Crohn's Disease

Medical management is the cornerstone of treating GCD. This review analyzed 20 case reports and series discussing various management strategies and therapeutic responses for this condition. Corticosteroids, widely recognized for their efficacy in CD, were frequently employed, with 14 studies utilizing them to treat GCD. While corticosteroids, immunomodulators, and TNF-α inhibitors are commonly employed with notable improvements, outcomes are often enhanced by combination therapies. Innovative approaches such as carbon laser therapy and stem cell transplants demonstrate significant potential. Surgical interventions are reserved for refractory cases or complications, emphasizing the challenges of managing this condition effectively. The frequency and percentage distribution of the various treatment modalities used in the reviewed cases are summarized in Supplementary Table 3.

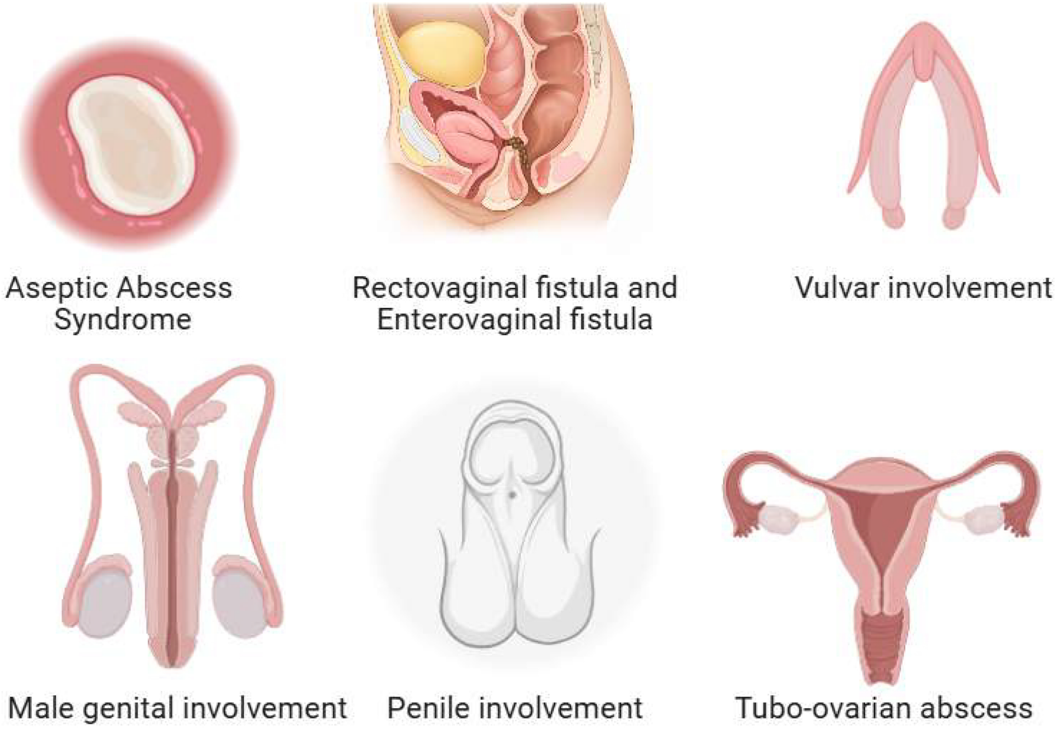

Figure 3. The genital Crohn's complications.

Corticosteroids (Topical, Oral, or Intravenous Formulations)

Topical corticosteroids showed mixed results. A study reported no improvement,[24] while another found that fluocinolone (twice daily for 2 weeks) combined with adalimumab (40 mg) led to symptom relief.[25] Another study reported the use of clobetasol propionate for vulvar CD.[26] However, GI symptoms flared within a month of treatment, suggesting the limited efficacy of topical therapies alone in addressing the systemic nature of the disease.

A study reported that mild and moderate potency topical corticosteroids were ineffective, but potent and very potent corticosteroids showed a good clinical response. Additionally, oral corticosteroids (median dose prednisone 20 mg) produced significant clinical improvement in vulvar symptoms in 13 patients.

Oral corticosteroids, a mainstay in treating intestinal CD, were also used for genital manifestations, though complete resolution was rarely achieved.[27] Oral corticosteroid use was reported in 8 studies with varying results, including 2 studies that noted no symptom resolution.[28] Complete resolution was documented in 1 study with prednisolone 60 mg daily as monotherapy.[29] The second study described significant improvement with intravenous prednisolone, leading to the resolution of skin lesions within 1 week. The patient was subsequently transitioned to oral prednisolone (30 mg daily) over 2 months, maintaining clinical remission.[30] Another case study observed initial symptom resolution with oral prednisolone doses ranging from 50 to 200 mg/day for anogenital CD, although 72% of patients experienced symptom relapse during dose tapering. Additionally, 3 studies highlighted the effectiveness of combining oral prednisolone (40 mg daily) with immunomodulatory agents such as methotrexate (7.5 mg), ustekinumab, or adalimumab. These findings emphasize the potential benefits of combination therapy, particularly in cases resistant to corticosteroid monotherapy.

Tumor Necrosis Factor-Alpha Inhibitors

A total of 9 studies support the use of TNF-α inhibitors for GCD, with most demonstrating symptom improvement or resolution. In a case series,[30] 6 patients were treated with infliximab or adalimumab for genital lymphedema, and 3 reported a reduction in symptoms. Vagianos et al[31] described temporary symptom relief with TNF-α inhibitors, while 7 studies noted overall symptom improvement following treatment with these agents. Drumond et al[32] documented complete symptom resolution in patients treated with infliximab at an induction dose of 5 mg/kg intravenously at weeks 0, 2, and 6, followed by a maintenance dose of 10 mg/kg every 8 weeks. This treatment was used in combination with carbon laser therapy. A total of 3 studies reported symptom improvement with TNF-α inhibitors after failure of other treatments, including topical and oral corticosteroids, antibiotics, and immunosuppressants such as azathioprine.[25,28,33] In these cases,[25] azathioprine was administered at 40 mg every other week during induction and 40 mg weekly as maintenance therapy, while infliximab was administered as infusions.[28] In 1 case report,[34] resolution of symptoms was achieved after 2 years of regular adalimumab therapy at

40 mg weekly, following an initial response to oral corticosteroids for GI symptoms. TNF-α inhibitors were used in dual therapy regimens with azathioprine, with symptom improvement observed when infliximab was switched to adalimumab.[35] In another case study, adalimumab was used in 7 patients; it was found to be effective in 4 patients. Treatment was stopped due to primary treatment failure in 2 patients and secondary treatment failure in 1 patient, with no documented adverse effects. The duration of treatment was 8 months to 6 years with weekly or fortnightly doses of 40 mg. Another case describes a 6-year-old child with penile edema who displayed incomplete resolution on oral infliximab.

Immunosuppressants (Azathioprine, Methotrexate, Mycophenolate Mofetil)

Immunosuppressants are frequently used in combination with TNF-α inhibitors and corticosteroids to treat GCD. Methotrexate at a dose of 7.5 mg[36] and azathioprine at doses ranging from 50 mg[37] to 200 mg[34] were reported to aid in symptom resolution. A case described symptom resolution with a mean azathioprine dose of 131.4 mg/day (range: 50–200 mg/day) and a treatment duration of 18.7 months (range: 5–46 months).[30] However, mycophenolate mofetil was discontinued in patients with CD due to side effects. Not all cases responded to immunosuppressant therapy. No symptom resolution was reported in 2 studies after a 6-week course of azathioprine.[33,38]

Another case showed variable responses with azathioprine with a daily treatment dose of 50–200 mg. However, all patients who received methotrexate for 1–6 years at a weekly dose of 10–25 mg stopped the drug due to either secondary failure and side effects or primary failure.

Aminosalicylates (Mesalazine, Sulfasalazine)

Only 1 study reported the use of oral aminosalicylate (mesalazine 1 g thrice daily) in combination with azathioprine, which resulted in the resolution of symptoms.[37] Mesalazine (2 g/day for 6 years) was used in only 1 patient and was found to be effective.

Novel Biologic Therapies: Ustekinumab

The use of ustekinumab in combination with oral prednisolone (40 mg) was described in 1 study.[38] The treatment began with an intravenous infusion of ustekinumab at 390 mg following the failure of anti-TNF-α therapy. The patient was then transitioned to subcutaneous injections of ustekinumab (90 mg every 8 weeks), which led to an almost complete resolution of symptoms.

Janus Kinases Inhibitors: Upadacitinib

Upadacitinib may play an important role in the treatment of refractory CD after the failure of biologic therapy and/or ustekinumab. In this study, an induction dose of upadacitinib (45 mg daily) and a maintenance dose of 15 mg daily with 5 mg prednisone were used to treat both the patient's CD-related colitis and his penile and scrotal inflammation, showing a dramatic response.

In another case study, a 33-year-old male patient reported active perianal disease and persistent painful scrotal swelling despite prior treatment with risankizumab and secondary loss of response to anti-TNF agents. The patient was transitioned to upadacitinib therapy, which involved an induction regimen of 45 mg once daily for 12 weeks, followed by a maintenance dose of 30 mg once daily. The patient experienced rapid improvement in scrotal swelling and complete resolution of genital symptoms.

Antibiotics

A total of 7 case reports mentioned the use of antibiotics in treating anogenital CD. These included doxycycline (100 mg once daily), co-trimoxazole (960 mg once daily or 480 mg twice daily), co-amoxiclav (375 mg thrice daily), clindamycin (250 mg once daily), trimethoprim (200 mg), metronidazole, erythromycin, and ciprofloxacin.[39] The study reported complete resolution of lesions using intravenous piperacillin, tazobactam, and metronidazole.[40] In 2 cases,[30,31] antibiotics were reported to be used in 84% of patients with anogenital CD, primarily to manage secondary cellulitis or asymptomatic, afebrile urinary tract infections. However, 4 studies reported no improvement in symptoms following antibiotic therapy.[28,29,33,40]

Stem Cell Therapy

García-Olmo et al[41] reported the innovative use of autologous stem cell transplantation for rectovaginal fistulas in patients with CD, which resulted in partial resolution of symptoms. Subsequent phase II and phase III trials demonstrated complete resolution, while allogeneic trials showed no rejection or adverse events. However, fistula closure was not achieved in these cases. Dozois et al[42] reported on the STem cells On Matrix Plugs phase 1 clinical trial aimed to evaluate the safety and feasibility of a novel stem cell-based treatment for refractory perianal CD. The treatment involved delivering autologous adipose-derived mesenchymal stem cells onto a bioabsorbable fistula plug in patients with complex CD perianal fistulas who had failed conventional therapies. In total, 20 patients (mean age 36 years) were treated with stem cell-loaded plugs. Complete clinical healing occurred in 14 of 18 patients at 6 months and 13 of 17 patients at 12 months. Furthermore, an MRI response was observed in 12 of 18 patients at 6 months. The trial showed that stem cell-loaded plugs can safely and effectively deliver cell-based therapy for patients with single-tract fistulizing perianal CD. However, the study was limited by its small sample size and restrictive inclusion criteria.

Carbon Laser Therapy

Carbon laser therapy has been explored as a treatment option for GCD, showing promising results in certain cases. A

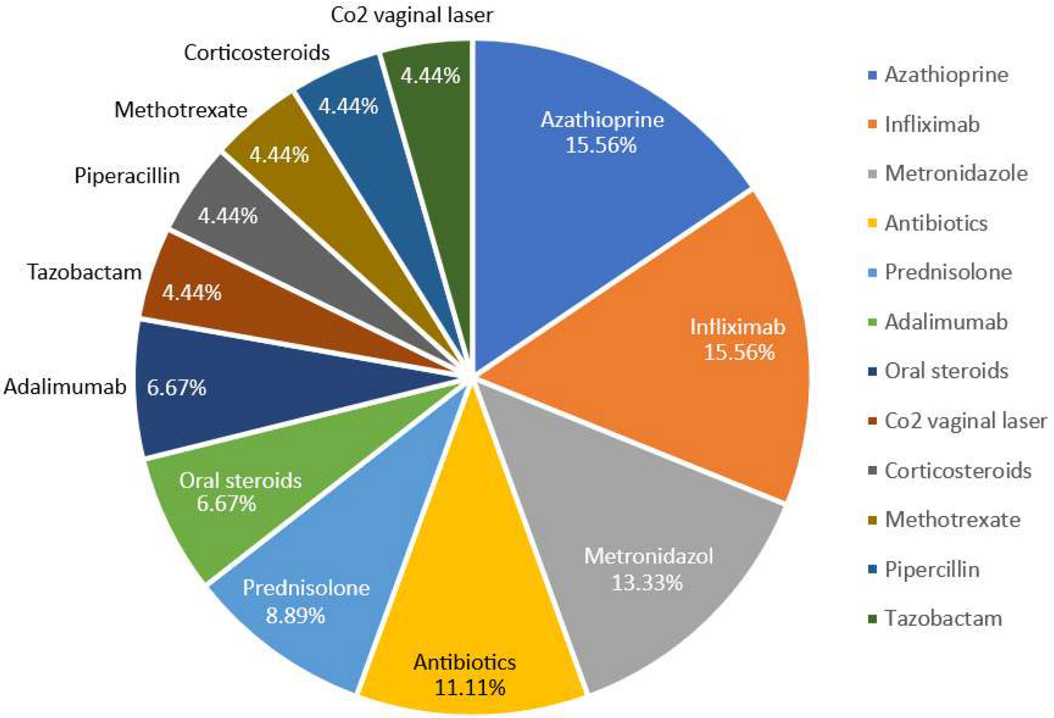

Figure 4. The percentage distribution of the different treatments for GCD.

cohort of patients underwent 5 sessions of carbon laser therapy,[32] with some reporting significant improvement in symptoms. While a subset of patients experienced partial symptom relief, others achieved complete remission following the therapy, highlighting its potential effectiveness in managing resistant lesions associated with CD. Successful treatment with a CO2 laser for vulvar lymphangiectasia was achieved in 1 patient. Additionally, patients receiving carbon laser therapy were often cotreated with infliximab, an anti-TNF-α agent. The infliximab regimen included an induction phase of 5 mg/kg administered intravenously at weeks 0, 2, and 6, followed by maintenance therapy at a dose of 10 mg/kg every 8 weeks.

Surgical Interventions in Crohn's Disease

Surgical intervention is often considered in cases of GCD when medical therapy fails to resolve lesions or when specific complications arise.[31] reported surgical management in 7 patients who did not achieve resolution with medical treatments. The procedures included detaching the inflamed bowel segment and suturing the fistula orifice. In cases where abscesses were present, codrainage was also performed, and all patients experienced resolution of symptoms without postoperative complications. Extensive perineal debridement and deroofing to healthy tissue is another surgical technique used in perianal CD, allowing the wound to heal via secondary intention.[43] Follow-up after 2 weeks showed good healing progress, with no need for additional surgical interventions. In another case, surgery was performed in 4 patients. Excision of swollen labia minora separated from the labia majora was performed in 3 patients, while excision of pedunculated, edematous tissue tags was performed in 1 patient. All the surgical procedures were successful. Given the impaired wound healing associated with CD and the potential for mutilating surgical outcomes, surgery should be reserved for specific scenarios: failure of medical therapy with significant quality-of-life impairment, debridement or drainage of vulvar abscesses, and resection of hypertrophic or unsightly lesions.

A summary of treatment options is discussed in Supplementary Table 4. The percentage distribution of the different treatments for GCD in our case report review is displayed in Figure 4.

Supplementary Materials

Material associated with this article can be found, in the online version, at https://doi:10.1016/j.gastha.2026.100918.

References

1. Cockburn E, Kamal S, Chan A, et al. Crohn's disease: an update. Clin Med 2023;23(6):549–557.
2. Danzi JT. Extraintestinal manifestations of idiopathic inflammatory bowel disease. Arch Intern Med 1988;148(2):297–302.
3. Shields BE, Richardson C, Arkin L, et al. Vulvar Crohn disease: diagnostic challenges and approach to therapy. Int J Women's Dermatol 2020;6(5):390–394.
4. Feller ER, Ribaudo S, Jackson ND. Gynecologic aspects of Crohn's disease. Am Fam Physician 2001;64(10):1725–1728.

5. Donaldson LB. Crohn's disease: "its gynecologic aspect". Am J Obstet Gynecol 1978;131(2):196–202.
6. Madnani NA, Desai D, Gandhi N, et al. Isolated Crohn's disease of the vulva. Indian J Dermatol Venereol Leprol 2011;77:342–344.
7. Dederichs F, Iesalnieks I, Sladek M, et al. Genital granulomatosis in male and female patients with Crohn's disease: clinical presentation and treatment outcomes. J Crohns Colitis 2018;12(2):197–203.
8. Andreani SM, Ratnasingham K, Dang HH, et al. Crohn's disease of the vulva. Int J Surg 2010;8(1):2–5.
9. Honap S, Meade S, Spencer A, et al. Anogenital Crohn's disease and granulomatosis: a systematic review of epidemiology, clinical manifestations, and treatment. J Crohns Colitis 2022;16(5):822–834.
10. Barret M, de Parades V, Battistella M, et al. Crohn's disease of the vulva. J Crohns Colitis 2014;8(7):563–570.
11. Tonolini M, Villa C, Campari A, et al. Common and unusual urogenital Crohn's disease complications: spectrum of cross-sectional imaging findings. Abdom Imaging 2013;38(1):32–41.
12. Kane S. Urogenital complications of Crohn's disease. Am J Gastroenterol 2006;101(12 Suppl):S640–S643.
13. Hartmann KA, Lerand SJ, Jay MS. Tubo-ovarian abscess in virginal adolescents: exposure of the underlying etiology. J Pediatr Adolesc Gynecol 2009;22(3):e13–e16.
14. Slaney G, Muller S, Clay J, et al. Crohn's disease involving the penis. Gut 1986;27(3):329–333.
15. Trefond L, Frances C, Costedoat-Chalumeau N, et al. Aseptic abscess syndrome: clinical characteristics, associated diseases, and up to 30 years' evolution data on a 71-patient series. J Clin Med 2022;11(13):3669.
16. Aberumand B, Howard J, Howard J. Metastatic Crohn's disease: an approach to an uncommon but important cutaneous disorder. Biomed Res Int 2017;2017:8192150.
17. Bhoyrul B, Lyon C. Crohn's disease of the vulva: a prospective study. J Gastroenterol Hepatol 2018;33(12):1969–1974.
18. Granese R, Calagna G, Morabito G, et al. Vulvar involvement in pediatric Crohn's disease: a systematic review. Arch Gynecol Obstet 2018;297(1):3–11.
19. Loftus EVJ. Clinical epidemiology of inflammatory bowel disease: incidence, prevalence, and environmental influences. Gastroenterology 2004;126(6):1504–1517.
20. De Cassan C, De Vroey B, Dussault C, et al. Successful treatment with adalimumab in a familial case of gastrointestinal Behcet's disease. J Crohns Colitis 2011;5(4):364–368.
21. Demir O, Yondem OZ, Doganay M. A neglected disease: hidradenitis suppurativa a rare cause of amyloidosis complicated with sepsis and renal failure: a case report. Case Rep Crit Care 2024;2024:4893040.
22. Reitsma W, Wiegman MJ, Damstra RJ. Penile and scrotal lymphedema as an unusual presentation of Crohn's disease: case report and review of the literature. Lymphology 2012;45(1):37–41.
23. Martínez F, Nos P, Benlloch S, et al. Hidradenitis suppurativa and Crohn's disease: response to treatment with infliximab. Inflamm Bowel Dis 2001;7(4):323–326.
24. Guest GD, Fink RL. Metastatic Crohn's disease: case report of an unusual variant and review of the literature. Dis Colon Rectum 2000;43(12):1764–1766.
25. O'Farrell C, Roberts A, Riera Canales C, et al. Genital and intertriginous rashes refractory to antimicrobial treatments: have you thought about Crohn's disease? Case Rep Pediatr 2021;2021:5578810.
26. Bouzidi H, Chakiri R, Amraoui N, et al. Crohn's disease presenting as vulvar edema in a 15-year-old girl. J Dermatol Case Rep 2014;8(3):75–77.
27. Benchimol EI, Seow CH, Steinhart AH, et al. Traditional corticosteroids for induction of remission in Crohn's disease. Cochrane Database Syst Rev 2008;2008(2):CD006792.
28. Wells LE, Cohen D. Delayed diagnosis of vulvar Crohn's disease in a patient with no gastrointestinal symptoms. Case Rep Dermatol 2018;10(3):263–267.
29. Dudley AG, Fox JA, Reyes-Múgica M, et al. Penoscrotal edema and purpura in a 12-year-old boy: a case report and review of causes. J Pediatr Urol 2012;8(5):e47–e50.
30. Alexakis C, Gordon K, Mellor R, et al. Ano-genital granulomatosis and Crohn's disease: a case series of males presenting with genital lymphoedema. J Crohns Colitis 2017;11(4):454–459.
31. Vagianos C, Malgarinos G, Spyropoulos C, et al. Enterovesical fistulas in Crohn's disease: a case series report and review of the literature. Int J Surg Case Rep 2017;41:477–480.
32. Drumond DG, de Condé CMS, da Chebli JMF, et al. Combined clinical and radiological remission of rectovaginal fistulas using fractional CO2 vaginal laser: a case series and medium-term follow-up. BMC Res Notes 2023;16(1):371.
33. Al Yacoub R, Brown K, Ladna M. Successful multidisciplinary treatment of the rare pathology of vulvar Crohn's disease. BMJ Case Rep 2023;16(10):e256343.
34. Garny de La Rivière C, Caudron A, Heyman B, et al. [Genital lymphedema associated with Crohn's disease]. La Rev Med Interne 2015;36(4):291–293.
35. Sethuraman A, Kontaki E, Gaynor E, et al. P206 paediatric genital Crohn's disease: presentation and treatment, a combined case series. J Crohns Colitis 2021;15(Supplement_1):S267–S268.
36. Gupta SK, Khalid A. Extraintestinal manifestation of Crohn's disease in an 11-year-old girl: a rare case report with review. Egypt J Dermatol Venereol 2024;44(1):55–58.
37. Kim S, Won YB, Seo SK, et al. Vulvar Crohn's disease in an adolescent diagnosed after unsuccessful surgical treatment. BMC Womens Health 2021;21(1):316.
38. Stoleru G, Robbins G, Papadimitriou JC, et al. Rapid resolution of vulvar Crohn's disease with ustekinumab. ACG Case Reports J 2020;7(8):e00452.
39. Bousvaros A, Schmidt BAR, Kurtz M. Treatment of genital crohn's disease with upadacitinib in a male child: a case report. Gastroenterol Hepatol (NY) 2023;19(7):401–403.
40. Ramji AN. Isolated vulvo-vaginal Crohn's disease: case report of the rare entity. Int Surg J [Internet] 2019;6:1400–1404.
41. García-Olmo D, García-Arranz M, Herreros D, et al. A phase I clinical trial of the treatment of Crohn's fistula by adipose mesenchymal stem cell transplantation. Dis Colon Rectum 2005;48(7):1416–1423.
42. Dozois EJ, Lightner AL, Dietz AB, et al. Durable response in patients with refractory fistulizing perianal Crohn's disease using autologous mesenchymal stem cells on a

dissolvable matrix: results from the phase I stem cell on matrix plug trial. Dis Colon Rectum 2023;66(2):243–252.
43. Moyes LH, Glen P, Pickford IR. Perineal metastatic Crohn's disease: a case report and review of the literature. Ann R Coll Surg Engl 2007;89(1):W1–W3.

Received May 12, 2025. Accepted March 5, 2026.

Correspondence:
Address correspondence to: Mohamed Elnaggar, MD, Hospital Medicine Department, Hartford Hospital, 80 Seymour Street, Hartford, Connecticut 06102. e-mail: Mohamed.elnaggar.md@gmail.com.

Conflicts of Interest:
The authors disclose no conflicts.

Funding:
The authors report no funding.

Ethical Statement:
The study did not require the approval of an institutional review board.

Data Transparency Statement:
Study materials will be made available.

Reporting Guidelines:
Reporting Guidelines were not applicable for this article type.

Gastro Hep Advances, Volume 5

Supplemental information

Unveiling Genital Crohn's Disease: Clinical Complications, Diagnosis, and Treatment, a Comprehensive Review of Case Reports

Bishoy Fahim, Mohamed Elnaggar, Mohamed Ayman Ebrahim, Shady Sapoor, Abdelrahman Helmy, Mahmoud Abd El-Nasser, Areeba Mariam Mehmood, Safia Elshennawy, Abdelrahman Sayed Al Komi, Laith Shakharteh, Mennatullah Ashour, Ismail Elkhattib, Mazen Gado, Esraa Soliman, Mohamed Abd El Aziz, and Hassan Ghoz

Supplementary Table 1: The distribution of the diagnostic modalities used across the included cases is summarized.

	Frequency	Percent
	1	2.0
barium meal	2	3.9
biopsy	12	23.5
blood/serology	1	2.0
clinical examination	1	2.0
colonoscopy	3	5.9
CT	10	19.6
endoscopy	4	7.8
MRI	12	23.5
radiographic	1	2.0
ultrasound/US	4	7.8
Total	51	100.0

Supplementary Table 2: Differential Diagnosis of Vulvar Crohn's Disease and Its Common Mimics.

**Categoy**	**Condition**	**Clinical Features**	**Distinguishing Features**
**Inflammatory**	Behçet's disease	Bipolar aphthous ulcers (oral/genital), recurrent ulcers, arthralgia, uveitis	Pathergy test positive; systemic inflammatory involvement
	Hidradenitis suppurativa	Painful nodules, abscesses, sinus tracts, and scarring in intertriginous areas	Follicular plugging and suppurative granulomas
	Sarcoidosis	Non-tender papules/plaques,	Non-caseating granulomas; systemic

		systemic symptoms (e.g., cough, fatigue)	organ involvement
	Pyoderma gangrenosum	Painful ulcers with undermined violaceous borders; associated systemic disease	Ulcerative lesions without bacterial cause
	Psoriasis	Well-demarcated, erythematous plaques with scaling; pruritus	Auspitz sign; silvery scales; nail pitting
	Atopic/Contact dermatitis	Pruritic erythematous rash after irritant/allergen exposure	Vesicles in acute phase; history of exposure

**Infectious**	Vulvovaginitis (fungal: C. albicans, bacterial: G. vaginalis, parasitic: T. vaginalis)	Vaginal discharge, pruritus, erythema, dysuria	Positive microscopy: yeast, clue cells, or motile trichomonads
	Other bacterial vulvitis (e.g., tuberculosis, actinomycosis, lymphogranuloma venereum)	Chronic non-healing ulcers, inguinal lymphadenopathy, systemic symptoms	Suggestive histology, specific bacterial culture or PCR
**Bacterial**	Tuberculosis (Mycobacterium tuberculosis)	Chronic non-healing ulcers, systemic symptoms (weight loss, fever)	Caseating granulomas on biopsy
	Staphylococcus aureus,		

	Streptococcus, Chlamydia trachomatis, Neisseria gonorrhoeae		
**Spirochete**	Syphilitic chancre (Treponema pallidum)	Solitary, painless ulceration; associated lymphadenopathy	Painless, clean-based ulcer with positive serology
**Viral**	Herpes simplex virus (HSV-2)	Painful vesicular lesions progressing to ulcers; recurrent episodes	Vesicles before ulceration; multinucleated giant cells (Tzanck)
	Ulcus vulvae acutum	Painful vulvar ulcers; viral prodrome (fever, URI symptoms)	Acute onset with systemic viral symptoms
	Condyloma acuminata (HPV)	Exophytic, wart-like papules/plaques; may be painful or	HPV-related histology: koilocytes and

		asymptomatic	papillomatosis
**Fungal**	Candidiasis	Erythematous rash, white plaques, satellite lesions, pruritus	Yeast forms and pseudo hyphae under microscopy
**Nutritional**	Zinc deficiency	Painful fissures, ulcers, poor wound healing; alopecia	Responds to zinc supplementation
**Malignant**	Squamous cell carcinoma	Chronic non-healing ulcer with indurated margins	Atypical squamous cells; invasive malignancy
	Vulvar intraepithelial	Pruritic, white/red patches or lesions	

	neoplasia		Dysplasia in epithelial layer
	Paget’s disease of the vulva	Erythematous, eczematous lesions; chronic, pruritic, and non-responsive to therapy	Paget cells: large pale cells in epidermis
**Other**	Genital lymphoedema	Chronic swelling, thickened skin, recurrent infections	History of trauma, obesity, or lymphatic obstruction
	Foreign-body reaction	Ulceration, granulomas, and inflammation; history of foreign material exposure	Foreign material identified in granulomas
	Vulvar edema	Swelling, thickened	History of radiotherapy,

	(post-radiotherapy, lymphedema, anasarca)	skin, peau d’orange appearance	lymphatic obstruction, systemic edema
	Epidermoid carcinoma	Chronic ulceration or mass; pain; induration; bleeding	Atypical squamous cells, invasion into deeper tissue

Supplementary Table 3: The frequency and percentage distribution of the various treatment modalities used in the reviewed cases are summarized.

	Frequency	Percent
adalimumab	3	6.7
antibiotics	5	11.1
azathioprine	7	15.6
CO2 vaginal laser	2	4.4
corticosteroids	2	4.4
infliximab	7	15.6
methotrexate	2	4.4
metronidazole	6	13.3
oral steroids	3	6.7
piperacillin	2	4.4
prednisolone	4	8.9
tazobactam	2	4.4
Total	45	100.0

Supplementary Table 4: A summary of treatment options

Medication Class	Drug	Dosage/Regimen	Notes
Corticosteroids	Topical Fluocinolone	Twice daily for two weeks	Combined with adalimumab (40 mg) for symptom relief. Limited evidence suggests mixed efficacy; one study reported no improvement, while another noted symptom relief when combined with systemic therapy.
	Topical Clobetasol	Applied once or twice daily (exact frequency not specified)	Limited efficacy; gastrointestinal symptoms flared within one month
	Oral Prednisolone	60 mg daily (monotherapy)	Complete resolution reported in one study, though rare.
	Oral Prednisolone	50–200 mg/day	Initial symptom resolution was observed, but 72% of patients experienced relapse during dose tapering.

	Oral Prednisolone	40 mg daily	Often combined with immunomodulators (e.g., methotrexate 7.5 mg weekly, ustekinumab, or adalimumab) for enhanced efficacy in refractory cases.
	IV Prednisolone	High-dose IV (exact dose not specified)	Transitioned to oral prednisolone (30 mg daily) over two months, maintaining clinical remission.
TNF-α Inhibitors	Infliximab	Induction: 5 mg/kg IV at weeks 0, 2, and 6; Maintenance: 10 mg/kg every 8 weeks	Combined with carbon laser therapy in some cases. Demonstrated complete symptom resolution in patients with refractory disease.
	Adalimumab	40 mg weekly or every other week	Used alone or in combination with azathioprine (40 mg every other week during induction, 40 mg weekly as maintenance).
Immunos uppressan	Azathioprine	50–200 mg/day	Mean dose: 131.4 mg/day; treatment duration: 18.7 months (range: 5–46 months).

ts			Effective in combination with TNF-α inhibitors or corticosteroids.
	Methotrexate	7.5 mg weekly	Used in combination with oral prednisolone (40 mg daily) for refractory cases.
	Mycophenolate Mofetil	Usually discontinued due to side effects.	Not recommended due to lack of efficacy and adverse events in Crohn's disease.
Aminosalicylates	Mesalazine	1 g three times daily	Only one study reported its use in combination with azathioprine, resulting in symptom resolution. Limited evidence for genital Crohn's disease.
Biologic Therapies	Ustekinumab	Induction: 390 mg IV; Maintenance: 90 mg subcutaneously every 8 weeks	Used as co-therapy with oral prednisolone (40 mg daily) after failure of anti-TNF-α therapy. Led to almost complete resolution of symptoms.
Antibiotics	Doxycycline	100 mg once daily	Used for secondary infections (e.g., cellulitis,

			UTIs). Limited efficacy in treating primary genital Crohn's lesions.
	Co-trimoxazole	960 mg once daily or 480 mg twice daily	Commonly used for secondary infections.
	Co-amoxiclav	375 mg three times daily	Commonly used for secondary infections.
	Clindamycin	250 mg once daily	Commonly used for secondary infections.
	Trimethoprim	200 mg	Commonly used for secondary infections.
	Ciprofloxacin	500 mg twice daily	Commonly used for secondary infections.
	Piperacillin/Tazobactam	IV (combined with metronidazole)	Complete resolution of lesions reported in one case. Primarily used for severe infections.
Stem Cell Therapy	Autologous Stem Cells	Delivered via bioabsorbable fistula plug	Phase I trial (STOMP) showed complete clinical healing in 14/18 patients at 6 months and 13/17 at 12 months. MRI response observed in 12/18 patients at 6 months. Safe

			and effective for refractory cases.
Carbon Laser Therapy	Carbon Laser	Five sessions (frequency not specified)	Combined with infliximab (5 mg/kg induction, 10 mg/kg maintenance). Partial to complete symptom relief reported in some cases.
Surgical Interventions	Various Procedures	Detachment of inflamed bowel, fistula suturing, abscess drainage, debridement	Reserved for refractory cases or complications (e.g., abscesses, hypertrophic lesions). Extensive perineal debridement and deroofing to healthy tissue also reported, with good healing progress.
